# Nature-Based Equity: An Assessment of the Public Health Impacts of Green Infrastructure in Ontario Canada

**DOI:** 10.3390/ijerph18115763

**Published:** 2021-05-27

**Authors:** Vidya Anderson, William A. Gough, Branka Agic

**Affiliations:** 1 Climate Lab, Department of Physical and Environmental Sciences, University of Toronto Scarborough, Toronto, ON M1C 1A4, Canada; william.gough@utoronto.ca; 2 Department of Physical and Environmental Sciences, University of Toronto Scarborough, Toronto, ON M1C 1A4, Canada; 3 Centre for Addiction and Mental Health (CAMH), Toronto, ON M6J 1H4, Canada; branka.agic@camh.ca; 4 Dalla Lana School of Public Health, University of Toronto, Toronto, ON M5T 3M7, Canada

**Keywords:** adaptation, climate change, community gardens, environmental health, food security, green roofs, growing roofs, rooftop farms, sustainable development, urban agriculture

## Abstract

The built environment is a physical determinant of health essential to the planning and development of a more equitable society. Communities face growing challenges due to environmental stressors such as climate change, with vulnerable communities experiencing a disproportionate burden of adverse health outcomes. The interdependencies between urban planning and public health outcomes are inextricable, with respect to improving access to healthier built environments for vulnerable and marginalized groups. Widespread implementation of nature-based solutions, such as green infrastructure, provides a multi-functional strategy to support sustainable development, increase climate resilience, enhance ecological connectivity, and create healthier communities. A Health Equity Impact Assessment presents the findings of a participatory research study utilizing key informant interviews of public health unit professionals (eight) and a survey of green infrastructure volunteers and workers (36) on the impact of green infrastructure on individual and community mental and physical well-being, service use, and perceived unmet needs, using Ontario, Canada as a case study. Study findings indicate that where green infrastructure is both productive and publicly accessible, the benefits were significant for vulnerable populations. These benefits include increased social connectivity, skills development, and food security. Green infrastructure could be a viable strategy to address environmental stressors, improve health equity, and support localization of the UN Sustainable Development Goals (SDGs).

## 1. Introduction

Nature-based solutions have been defined by the International Union for Conservation of Nature (IUCN) as “actions to protect, sustainably manage, and restore natural or modified ecosystems, that address societal challenges effectively and adaptively, simultaneously providing human well-being and biodiversity benefits” [1]. Nature-based solutions describe the five categories of ecosystem-based approaches which include green infrastructure [1,2,3,4,5,6]. Widespread implementation of green infrastructure provides a nature-based solution to alleviate environmental stressors from climate change and to perform the dual functions of climate change mitigation and adaptation simultaneously [4,5,6,7,8]. Green infrastructure can function as a complex form of adaptation that both minimizes the most harmful effects of climate change on human health and mitigates greenhouse gas (GHG) emissions that cause climate change.

**Figure 1:**
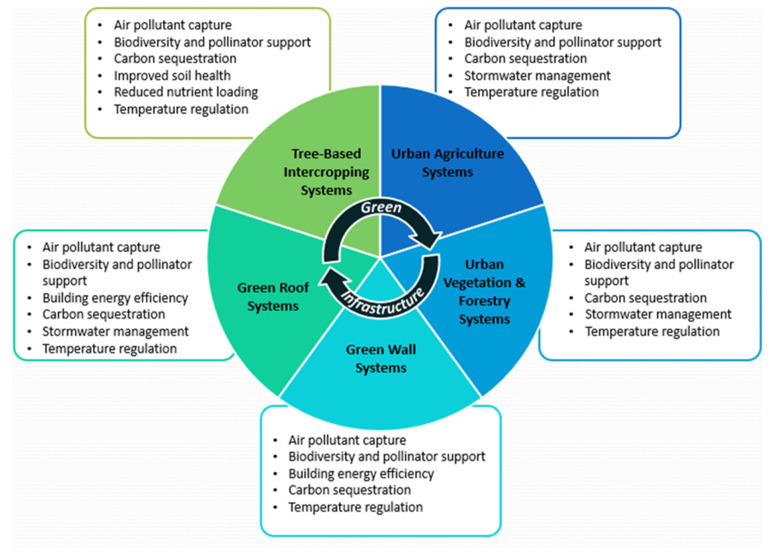
Green infrastructure form and function [4,5,7].

While environmental stressors resulting from climate change affect everyone, populations that are elderly, very young, chronically ill or physically impaired, lower income, socially disadvantaged, and marginalized are disproportionately affected [9,10,11]. Key environmental stressors include rising temperatures, flooding and extreme weather events, air pollution, drought, range expansion for disease vectors, and stratospheric ozone depletion [9,10,12,13,14,15]. Vulnerable populations are disproportionately exposed to these stressors by virtue of circumstance. Climate change can exacerbate the social determinants of health in vulnerable populations. The application of green infrastructure provides a mechanism for addressing environmental stressors resulting from climate change, with unique characteristics and multiple co-benefits such as biodiversity and pollinator support, building energy efficiency, and stormwater management that can be leveraged if strategically applied. There are common functions shared, as illustrated in Figure 1, while others are exclusive to particular applications.

Green infrastructure is defined as a series of interconnected networks of natural and constructed green space which provide various ecosystem services. Figure 1 shows the different applications of green infrastructure categorized into five areas: green roofs, green walls, urban vegetation and forestry, urban agriculture systems, and tree-based intercropping systems [4,5,6,7,8].

The application of green infrastructure has demonstrated human health benefits. Green infrastructure has a moderating effect on temperature, providing cooling capacity, and reducing the urban heat island (UHI) effect. For example, green roofs have been shown to be effective in reducing UHI effect and associated health risks [16]. Green infrastructure can improve respiratory health outcomes from extreme heat and air pollution [17,18,19,20,21,22,23,24]. Green infrastructure improves air quality through air pollutant and particulate capture. Green-roofing and green wall technologies can reduce air pollutant concentrations and provide urban cooling [4,8,25,26]. Urban vegetation and forestry have also been shown to be effective in reducing air pollution from nitrogen dioxide [4,24]. Green infrastructure is also a highly effective stormwater management tool that reduces flood risk and improves water quality [27,28,29]. In addition, there is growing recognition that green infrastructure, land use development patterns, and rising temperatures behave as barriers to or conduits for disease amplification and spread in human, domestic animal, and wildlife populations [30,31,32,33,34]. Green infrastructure can improve biodiversity by providing habitat and increasing connectivity between landscape networks. This is essential to reducing risk of infectious disease spread by providing habitat for vector and zoonotic reservoir populations [34].

Green infrastructure also provides multiple environmental and human health co-benefits that extend beyond climate change mitigation and adaptation. Evidence shows that green infrastructure can have positive impacts on the social determinants of health, in addition to supporting physical and psychological human health benefits [19]. The mental health benefits of green infrastructure have been demonstrated in the treatment of major depressive disorder and total mood disturbance [35,36,37]. In urban areas, green infrastructure is beneficial in the treatment of mental illness including anxiety, depression, and stress [38,39,40,41].

Green infrastructure supports healthy environments and provides ecosystem services fundamental to health and well-being including the provision of clean drinking water, food, breathable air, climate regulation, and natural resources for shelter, clothing, medicine, and energy production. There are also direct health co-benefits that result from the application of green infrastructure. Post-operative outcomes for patients recovering from surgery are improved by exposure to green infrastructure [42]. Human panel studies have shown that exposure to green infrastructure can reduce blood pressure, heart rate, and stress while increasing parasympathetic nerve activity and restoration, and improving immune response [43,44,45]. Green infrastructure also supports physical activity through the provision of multi-functional greenspace. Reduced mortality from cardiovascular, respiratory, and other causes have also been linked to residential green infrastructure across various cohort studies [46,47,48,49].

This paper presents the findings of a study exploring the impact of green infrastructure on individual and community mental and physical well-being, service use, and perceived unmet needs. This mixed-methods research study including both qualitative and quantitative elements, focused on the public health impacts of green infrastructure development within communities, and factors contributing to the community benefits of green infrastructure.

## 2. Methods

To support the development of this work, a Health Equity Impact Assessment (HEIA) was undertaken to identify potential unintended impacts of green infrastructure on vulnerable populations. Health Equity Impact Assessment tools (HEATs) are used widely across multiple jurisdictions including Australia, New Zealand, the United Kingdom, and Wales [50]. The Health Equity Impact Assessment process is also widely used and supported by the World Health Organization [51]. HEATs are decision support tools that help to identify how a policy or strategy will impact the health of population groups in different ways [52]. These tools provide a framework for approaching health inequities in a systematic way by identifying improvements in health equity as a goal, and providing a series of steps and evaluation questions, with a framework to achieve this goal. Similar assessment tools include health impact assessments, human health risk assessments, environmental risk assessments, and environmental impact assessments. The key difference between a HEAT and other types of assessments is that equity and the equitable distribution of health risks and benefits to a population are the key focus. HIAs focus on evaluating potential health effects of a policy or program on vulnerable populations but their focus is specifically on health effects [53]. A human health risk assessment (HRA) assesses the potential of adverse health effects from chemical or contaminant exposure [54]. Environmental risk assessments (ERAs) evaluate the potential of a development or an industrial process in harming the environment and the associated hazards and impacts [55,56]. Environmental impact assessments (EIAs) evaluate potential environmental impacts from a development or an industrial process, while also accounting for socioeconomic and health impacts [57]. HEATs increase policy coherence around health equity by assessing the potential consequences of decision making. This supports the policy development process and can be used to engage the most appropriate partners and stakeholders in dialogue [58]. These types of assessments are most efficient when meaningful stakeholder engagement is undertaken that incorporates wide representation to enable innovative collaboration and ensuring strategic and interconnected planning at the policy and program level [59]. HEATs help to evaluate a policy or program’s impact on health inequities and vulnerable populations [60].

**Figure 2:**
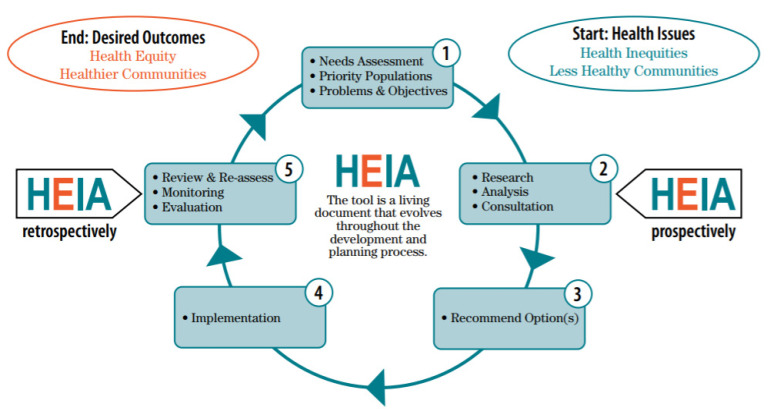
Health Equity Impact Assessment (HEIA) process [61].

The HEAT used to develop this assessment was developed by the Ontario Ministry of Health and Long-Term Care (MOHLTC) to identify and address potential unintended (positive or negative) impacts of a policy, program, or strategy on specific population groups as shown in Figure 2 [61]. The Ontario HEAT incorporates international evidence as well as input gathered during regional pilot projects [61]. The main purpose of this tool is to maximize positive impacts and reduce negative impacts by informing policy development that could potentially exacerbate health disparities between population groups [61].

**Figure 3:**
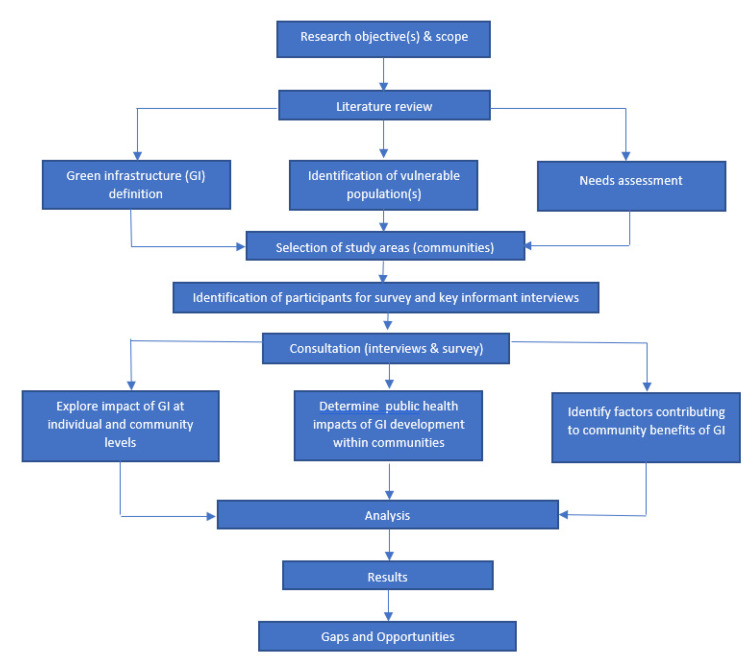
Research methodology process flow.

Because of the exploratory nature of this study, a mixed-methods research design using key informant interviews and surveys was employed as shown in Figure 3. Key informant interviews were employed to collect information from public health professionals in six local public health units with primary knowledge of the vulnerable populations within communities across Ontario, Canada and on the community users of green infrastructure who may belong to vulnerable population groups. Surveys were also employed to collect data from respondents at a productive and publicly accessible green infrastructure site to gain information and insights into the use and impact on the use and impact of green infrastructure on individual and community mental and physical well-being, service use, and perceived unmet needs. Prior to participant recruitment, an ethics review application and an accompanying research protocol were submitted for review to the Office of Research Ethics at University of Toronto on 3 August 2017. Approval to proceed with the research was granted on 1 October 2017.

### 2.1. Participants

Participants were selected using the snowball sampling method. Snowball sampling is also known as chain sampling, chain-referral sampling, or referral sampling. This is a non-random sampling technique, where existing study subjects recruit future subjects from among their acquaintances [62]. This sampling method is used where it is difficult to identify potential participants because the sample population for the study is limited to small subsections of the population [63].

**Figure 4:**
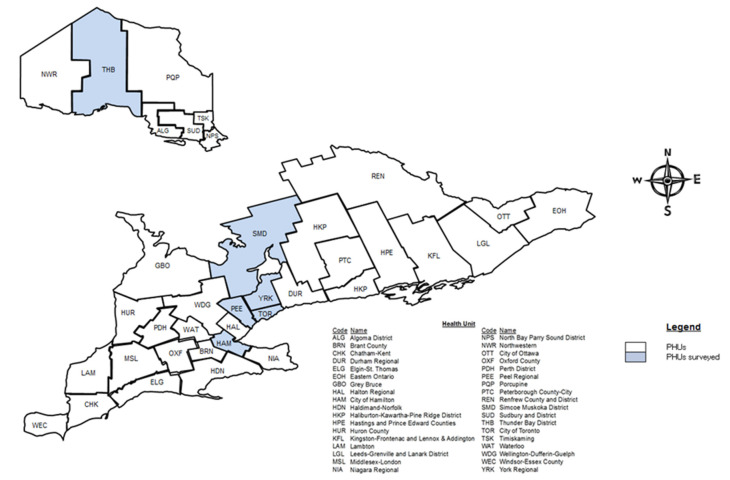
Map of public health units in Ontario, Canada. Shaded areas represent public health units surveyed [7].

Two groups of participants were identified for participation. The first group of participants identified, were public health professionals working in public health units across Ontario, Canada. The second group of participants identified were volunteers or workers on publicly accessible green infrastructure sites in Ontario, Canada. Participants from each group were asked to provide a narrative of their experience and relationship with green space and green infrastructure features in their community. All study participants consented for possible inclusion in the qualitative study. Ethics approval for the surveys and key informant interviews was granted by the Research and Ethics Board at the University of Toronto. Having two separate groups of participants allowed for a more fulsome analysis of the pertinent issues for each of these groups. The key informant interviews were conducted with six public health units including Toronto Public Health, Peel Region Public Health, York Region Public Health, City of Hamilton Public Health Services, Simcoe-Muskoka District Health unit, and Thunder Bay District Health unit. As shown in Figure 4, these six public health units were selected out of the 36 public health units across Ontario because they were representative of urban, suburban, rural, and remote communities, in addition to being representative of different types of built form. The selected public health units were contacted by email in October 2017 followed by a telephone call. Inclusion criteria for participating in the survey required individuals to be responsible for or have experience with health intervention activities related to environmental health, with a particular focus on healthy built environments. This is an identified function within public health units across Ontario, Canada [64]. Appropriate individuals who were identified are responsible for developing public health policy and programs within public health units. Individuals were asked to identify other potential participants specifically in the environmental health area, from amongst their colleagues who may have had experience with or knowledge of green infrastructure development and public health impacts within their communities. Although the age and gender of participants were not requested, interview participants included both men and women above eighteen years of age. All the public health units who were invited, chose to participate in the key informant interviews.

The survey was distributed in October 2017 to 36 participants who met the inclusion criteria of being directly engaged in planting and maintenance activities on green roofs as a worker or a volunteer. This sample size is consistent with survey methodology for this type of research [65,66,67,68]. The response rate for this survey was 100 percent. Survey participants included sixteen men, eighteen women, and two transgendered persons above eighteen years of age. The Carrot Green Roof and Community Garden managed by the Seeds of Hope Foundation, in Toronto, Ontario, Canada was selected as a study site because it is an example of productive and publicly accessible green infrastructure containing four different types of green infrastructure. Permission was sought from the foundation to engage the workers and volunteers who maintain the Carrot Green Roof and Community Garden for participation in the survey. Surveys and consent forms were provided directly to interested participants. Additional participants were also identified by the Seeds of Hope Foundation and these individuals were provided with surveys and consent forms.

### 2.2. Data Collection

Data were generated from the key informant interviews and survey to reveal positive and negative impacts of green infrastructure development that could potentially exacerbate health disparities between population groups. Using the open-ended survey and key informant interviews, participants were asked to provide a narrative of their experience and relationship with green infrastructure features in their community. The surveys and key informant interviews were conducted in English. The key informant interview and survey questions were developed for the public health unit participants and the volunteers and workers from the Carrot Green Roof and Community Garden to enable a discrete analysis of the pertinent issues for each of these groups as public health professionals working with vulnerable populations in their health unit areas and with community users of green infrastructure who may belong to vulnerable population groups.

Key informant interviews were conducted with six local public health units (including Toronto Public Health, Peel Region Public Health, Simcoe-Muskoka District Health Unit, Thunder Bay District Health Unit, Hamilton Public Health, and York Region Public Health) responsible for health intervention activities related to environmental health and healthy built environments, across Ontario, Canada. These six public health units were selected for participation because of their differing size and geographic location. By interviewing these different public health unit locations, this study aimed to understand how climate change impacts different vulnerable populations in different areas. The key informant interview questions were provided to each of the six public health units electronically and the interviews were completed by telephone with eight key informants who volunteered to complete the survey. A total of six individuals were contacted within each public unit who shared the interview questions with two additional participants as shown in Table 1. The eight public health unit participants were asked to answer a series of six open-ended questions focused on identifying vulnerable populations within the local health unit area, awareness of green infrastructure elements within the community, and communication of green infrastructure benefits to community residents. Participants were asked to identify which populations were most vulnerable in their health unit areas and to describe the nature of vulnerability. Participants were also asked to identify green infrastructure elements in their health unit areas and if any of these elements were publicly accessible and productive (i.e., food producing). Additionally, participants were asked how they promoted the health benefits of green infrastructure to vulnerable populations and the general public. The key informant questions are available in [7].

**Figure 5:**
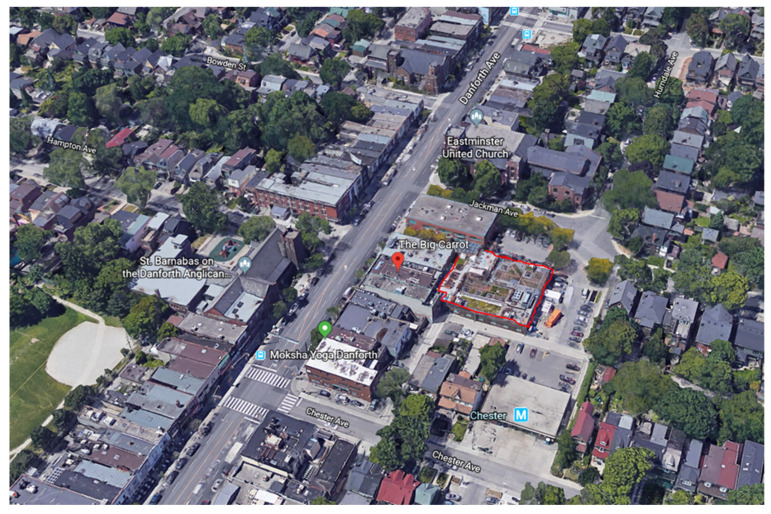
Map of Carrot Green Roof and Community Garden and surrounding area (courtesy of Google Earth).

The survey was distributed to 36 volunteers and workers directly engaged in planting and maintenance activities on the Carrot Green Roof and Community Garden in Toronto, with a minimum of 25 participants required for a viable study [65,66,67,68]. The Carrot Green Roof and Community Garden was selected as a functional example of productive (i.e., allows for the production of food) and publicly accessible green infrastructure. The Carrot Green Roof and Community Garden as shown in Figure 5, are managed by the Seeds of Hope Foundation which has five community homes offering a wide variety of resources and support programs, including two learning centres, three post-rehab recovery homes, and a women’s shelter. Residents from these various facilities built the 10,000 square foot Carrot Green Roof and Garden, in addition to ongoing planting and maintenance of the space. The space contains multiple applications of green infrastructure including a growing roof for various types of produce, a green roof, a herb and vegetable garden, and a green wall. The site is an urban learning hub that provides a wide variety of fresh produce including fruit, vegetables, herbs, and medicinal plants. The produce from this site was used by the residents from the various Seeds of Hope Foundation facilities within the city.

Volunteers and workers directly engaged in planting and maintenance activities on the Carrot Green Roof and Community Garden were asked a series of eleven multiple choice questions to quantify and qualify the nature of their usage of the site. Questions were focused on identifying participants’ access to green space and fresh food, opportunities for social interaction and skills development, and demographic profile [7]. A factor within the demographic profile is income. Within the survey, annual income was measured as either above or below $15 K CAD. The annual income amount of $15 K CAD was selected because it aligns with the annual basic income benefit amount established as part of the Basic Income Guarantee (BIG) program in Ontario, Canada. The BIG amount is based on the Low-Income Measure (LIM) defined by the Ontario Works (OW) Program and the Ontario Disability Support Program (ODSP). The LIM is a common income-based measure used to define poverty.

### 2.3. Data Analysis

Qualitative content analysis was undertaken through identification of common issues, similarities, and differences that emerged from the surveys and the key informant interviews. Coding was performed manually, and secondary analyses were stimulated by concepts that emerged in the data and from further literature review. These themes and patterns were related to the research questions to produce new knowledge on the topic, specifically related to the public health impacts of green space and green infrastructure development within communities.

### 2.4. Study Limitations

The sample size for this HEIA study was relatively small and limited to two groups of participants that included a group of key informant interviewees (8 persons) and a group of survey participants (36 persons). Survey participants were not a representative sample selected from a particular vulnerable population, but rather a group of people who either embody the characteristics or live under circumstances relevant to the issue being investigated. Due to the small sample size, statistical analysis of the findings was not viable.

## 3. Results

Analysis of the survey questionnaires and key informant interviews revealed key themes that underscored the importance of green infrastructure development that is both productive and publicly accessible. Four major themes emerged from this analysis including vulnerable populations; norms, attitudes, and beliefs about green infrastructure; green infrastructure access and usage; and relevant messaging.

### 3.1. Vulnerable Populations

Participating public health units identified key vulnerable populations within their health unit areas. Across all six public health units interviewed, vulnerable populations that were identified included seniors, young children, individuals of lower socioeconomic status (SES) with an annual income of less than $15 K CAD, individuals with chronic illness or disability, and those “underhoused or homeless, or living in a shelter, apartment, or basement dwellers with no renter’s insurance or access to air conditioning.” Within particular public health unit areas, certain populations were more vulnerable because of other risk factors. Vulnerable populations were identified by public health units through surveillance and epidemiology, in addition to the use of Geographic Information Systems (GIS) mapping. In the Simcoe-Muskoka district area, there were populations vulnerable because of geographical location. Within the health unit area, individuals living in the local floodplain were repeatedly impacted by flooding, and experienced periodic displacement and property damage. Those living in floodplain areas were individuals of lower SES with limited resources to manage these impacts.

In the Peel regional health unit area, individuals living in proximity to the Cooksville Creek and Port Credit areas were impacted by flooding and property damage because of the local geography. The majority of the watershed is impermeable due to the pattern of development and the aging stormwater infrastructure. In Caledon, which is a mix of agricultural lands and residential communities, “residents were vulnerable to drought as drinking water is well-based”. Residents of Brampton and Mississauga were vulnerable to vectorborne disease such as West Nile Virus and Lyme disease due to range proximity and expansion as result of development. Within the Thunder Bay District Health Unit area, Indigenous populations in particular, were identified as vulnerable because of social marginalization and lower SES. Survey participants from the Carrot Green Roof and Community Garden were individuals of lower socioeconomic status (SES) with an annual income of less than $15k CAD.

### 3.2. Norms, Attitudes and Beliefs about Green Infrastructure

Participating public health units had a shared understanding of the impacts of environmental stressors on human health and the populations who are most vulnerable. With respect to what green infrastructure is, interview participants had a shared understanding of its characteristics, but varying beliefs regarding its ability to act as an intervention. Findings revealed that public health unit participants held common beliefs about the public health impacts and community benefits of publicly accessible and productive green infrastructure such as green roofs, green walls, rooftop gardens, and community gardens.

The most commonly shared belief across all public health units about the benefits of green infrastructure application, was that it facilitates recreation and physical activity. Other discretely recognized benefits included shading and cooling to address urban heat island (UHI) effect and improvements to local air quality. Within certain health unit areas, other benefits of green infrastructure were recognized. In both the Simcoe-Muskoka district and Peel regional health unit areas, green infrastructure was recognized as beneficial for flood attenuation, stormwater management, and source water protection. Across the Hamilton, Simcoe-Muskoka and Thunder Bay district unit areas, green infrastructure was recognized as beneficial in supporting food security. In the Simcoe-Muskoka district health unit area, there was recognition that “green infrastructure is beneficial in building social cohesion and supporting mental health”.

There was common recognition across the six public health units that green infrastructure has public health benefits for local communities, but context was variable. The interdependencies between urban planning and development using green infrastructure and the capacity of its application to exacerbate or mitigate environmental impacts were not well understood.

### 3.3. Green Infrastructure Access and Usage

Across the six public health units, public access to green infrastructure varied. The most common applications of publicly accessible green infrastructure across health unit areas included urban forestry and vegetation in parks and nature trails, and community gardens. Of those applications, only community gardens were productive (i.e., allow for the production of food). In the Hamilton public health unit area, a large network of community gardens produced approximately 175K pounds of food supporting local food banks. This work was supported by the work of “well-mobilized volunteers and green infrastructure users of lower socioeconomic status (SES)”. The development and implementation of the community garden network was a strategic initiative developed by the Hamilton public health unit to increase food security and reduce the local carbon footprint. Within the Thunder Bay district health unit area, 27 community gardens and approximately 16 school gardens and greenhouses have been developed to support community food security.

**Figure 6:**
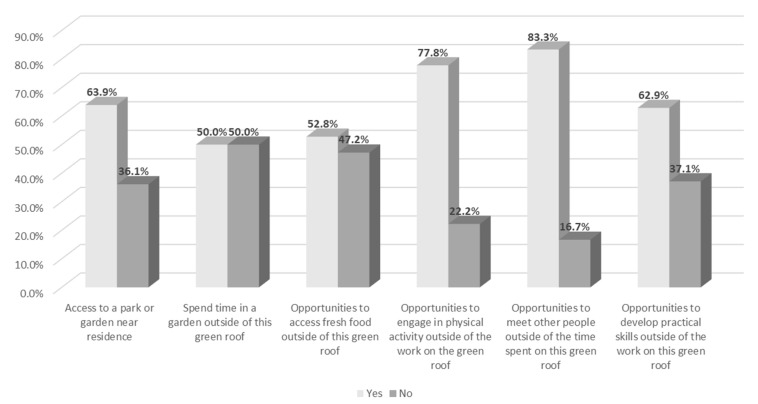
Summary of responses.

Of the survey participants directly engaged in planting and maintenance activities, 36 percent did not have access to green infrastructure or green space outside of the Carrot Green Roof and Community Garden and 50 percent did not spend time in a park, garden, or green space outside of this space. The green roof and garden also supported other important functions such as physical activity, social connectivity, and skills development. With the exception of planting and maintenance activities on the Carrot Green Roof and Community Garden, 22 percent of survey participants did not have other opportunities to engage in physical activity, 17 percent did not have other opportunities to meet or socialize with other people, and 37 percent did not have other opportunities to learn or develop practical skills as shown in Figure 6.

**Figure 7:**
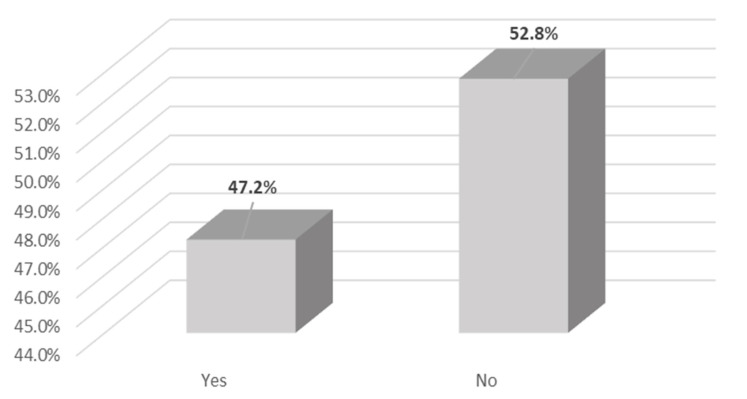
Percentage of green roof users with access to fresh food outside the Carrot Green Roof and Community Garden.

Perhaps the most revealing statistic is that 47 percent of the survey participants did not have opportunities to access fresh food outside of the Carrot Green Roof and Community Garden as shown in Figure 7.

**Figure 8:**
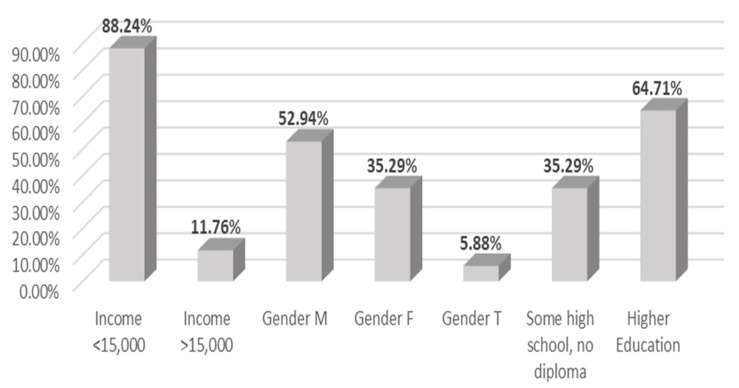
Demographic breakdown of users with no fresh food access outside of Carrot Green Roof and Community Garden.

Survey results showed that users of the green roof who did not have access to fresh outside of the Carrot Green Roof and Community Garden were members of vulnerable populations including those of lower income. Figure 8 illustrates the demographic breakdown of the green roof users with no access to fresh food outside the Carrot Green Roof and Community Garden, with 88 percent of these users having an annual income of less than $15k CAD.

### 3.4. Relevant Messaging

Although there was common recognition across participating public health units of the health benefits of green infrastructure for local communities, the application of green infrastructure and its capacity to mitigate environmental stressors were not homogeneously articulated. Recreation and physical activity were the most commonly publicized benefits of green infrastructure across public health units and information was made publicly available through active transportation initiatives such as nature trail strategies. Shade and cooling benefits of green infrastructure were also made publicly available, often in conjunction with heat and air quality management initiatives such as the Heat Warning and Information System and Air Quality Health Index in Ontario which have specific public messaging that references the benefits of green infrastructure elements such as urban forestry and vegetation. In the Hamilton and Thunder Bay district public health units, food security was a benefit communicated as evidenced by local food strategies. Other mechanisms available for public health units to communicate the benefits of green infrastructure included municipal and regional official plan reviews, and various healthy built environment strategies.

## 4. Discussion

Using the HEIA approach for this study has revealed unintended consequences and potential impacts of green infrastructure development in Ontario, Canada. This study has identified gaps and opportunities for equity-based improvements in the mainstream implementation of green infrastructure to improve health outcomes for all Ontarians. The findings confirm the beneficial public health impacts and community benefits of publicly accessible and productive green infrastructure including green roofs, green walls, rooftop gardens, and community gardens. Vulnerable populations face different challenges, depending on geography and infrastructure. This emphasizes the importance of widespread equitable green infrastructure development to mitigate and provide adaptive capacity to the human health impacts of climate change.

Across all public health unit areas, green infrastructure applications that met the criteria of being both productive and publicly accessible, were primarily limited to community gardens. Publicly accessible green infrastructure was focused on urban vegetation and forestry systems in the form of parks and trails. Where other types of green infrastructure such as green roofs systems or roof top gardens exist across health unit areas, most were not publicly accessible or productive. The results of this study also indicated that where green infrastructure is both productive and publicly accessible, the benefits were significant for vulnerable populations. These benefits include access to green space, increased social connectivity, skills development, and food security.

The Carrot Green Roof and Community Garden provided 36 percent of survey participants with access to green space that would not otherwise have been accessible to them. Additionally, 50 percent of participants did not spend time in a park, garden, or green space outside of this space, suggesting its significance in the provision of therapeutic benefits. With the exception of planting and maintenance activities on the Carrot Green Roof and Community Garden, 22 percent of survey participants did not have other opportunities to engage in physical activity, 17 percent did not have other opportunities to meet or socialize with other people, and 37 percent did not have other opportunities to learn or develop practical skills. These results support similar findings by Ward-Thompson et al. [69] that access to green space in neighborhoods with lower SES provided a sense of place belonging, and reduced feelings of social isolation. Additionally, urban publicly accessible green infrastructure may reduce the effects of stressors such as unemployment [40,69].

The food security benefits of productive green infrastructure cannot be overstated, especially for vulnerable populations within urban areas. This study revealed that 47 percent of the survey participants did not have opportunities to access fresh food outside of the Carrot Green Roof and Community Garden. Those who did not have access to fresh food outside of this green space were members of vulnerable populations including those of lower income with 88 percent having an annual income of less than $15k CAD.

Green infrastructure in the form of urban agriculture systems can reduce the pressures on conventional agriculture, improving food security when large-scale agricultural production is affected by weather variation [6]. These benefits are significant within the broader context of climate change. Urban agriculture systems can increase food security by reducing the food miles associated with conventional agriculture through localized food production and distribution from growing roofs to rooftop, terrace, backyard, and community gardens to food forests [6]. The primary aim of this mixed-methods research has been to understand the public health impacts of green infrastructure development within communities and what factors contribute to the community benefits of publicly accessible and productive green infrastructure such as green roofs, green walls, rooftop gardens, and community gardens. By undertaking a Health Equity Impact Assessment for green infrastructure, this study shows how green infrastructure behaves as a complex climate change and public health intervention that can moderate harms and capitalize upon beneficial opportunities for vulnerable populations who may be disproportionately affected by environmental stressors from a changing climate. This study has identified potential gaps and opportunities for equity-based improvements in the mainstream implementation of green infrastructure, with respect to food security and public access to green infrastructure. For the users of the Carrot Green Roof and Community Garden, this space has supported community mental and physical well-being. Well-being was supported by access to fresh food, opportunities to learn new skills and to spend time with other people, and access to green space.

The information from this research can be used to incorporate the community benefits of green infrastructure into public health policy, and to develop recommendations for making green infrastructure more productive and publicly accessible across Ontario, Canada. This information is beneficial for decision makers within:

- Public health units as it relates to environmental health and the impacts of the natural and built environment on population health including heat stress, air quality and food security;
- Municipal planning departments as it relates to new development, building retrofits and re-zoning including energy efficiency, stormwater management, and extreme weather events; and
- Provincial and federal government ministries as it relates to government priorities such as climate change mitigation and adaptation; increased food security; and improved air and water quality.

### Future Research

Since vulnerable populations are disproportionately exposed to the impacts of climate change by virtue of circumstance, future work of interest includes a cross-study to measure stress response using hair or saliva sampling withing specific green infrastructure settings to understand how this type of intervention can better address the disproportionate exposure of those who are vulnerable. It would be beneficial to partner with a public health unit and a charitable organization within a specific community to undertake such a study.

Additionally, it would be beneficial to undertake a HEIA study across multiple productive and publicly accessible green infrastructure sites to better understand how to reduce the pressures on conventional agriculture and improve food security and community resilience when large-scale agricultural production is affected by weather variation and other public health emergencies.

## 5. Conclusions

Environmental stressors resulting from climate change will continue to exacerbate health and social disparities. Vulnerable populations will experience these associated health impacts more acutely. The application of productive and publicly accessible green infrastructure is a climate change and public health intervention that can moderate harms and capitalize upon beneficial opportunities for vulnerable populations who are disproportionately affected by the changing climate. This study has identified potential gaps and opportunities for equity-based improvements in the mainstream implementation of green infrastructure to improve health outcomes. Green infrastructure optimizes the built environment by improving the urban microclimate. It also leverages other co-benefits including stormwater matter management; air pollution abatement; biodiversity and pollinator support; and enhanced food security. Green infrastructure offers a multi-faceted, nature-based solution to the challenges presented by different urban morphologies. Green infrastructure also provides multiple environmental and human health co-benefits that extend beyond climate change mitigation and adaptation. It has positive impacts on the social determinants of health, in addition to supporting physical and psychological human health benefits. This information can be used to inform change in green infrastructure development and land use planning policy. It also provides a foundation for cross-system integration between public health and municipal planning departments in land use planning decisions. This is fundamental for the development and implementation of effective and equitable green infrastructure.

## Figures and Tables

**Table 1:** Public health units interviewed.

Public Health Unit	Number Invited	Number Interviewed
Toronto Public Health	1	1
Peel Region Public Health	1	1
Simcoe-Muskoka District Health Unit	1	1
Thunder Bay District Health Unit	1	1
Hamilton Public Health	1	3
York Region Public Health	1	1

## Data Availability

The data presented in this study are available on request from the corresponding author. The data are not publicly available due to privacy reasons.
